# Prognostic Value of Computed Tomography: Measured Parameters of Body Composition in Primary Operable Gastrointestinal Cancers

**DOI:** 10.1245/s10434-017-5829-z

**Published:** 2017-03-21

**Authors:** Douglas Black, Craig Mackay, George Ramsay, Zaid Hamoodi, Shayanthan Nanthakumaran, Kenneth G. M. Park, Malcolm A. Loudon, Colin H. Richards

**Affiliations:** University Department of Surgery, Aberdeen Royal Infirmary, Aberdeen, UK

## Abstract

**Background:**

Previous reports suggest that body composition parameters can be used to predict outcomes for patients with gastrointestinal (GI) cancers. However, evidence for an association with long-term survival is conflicting, with much of the data derived from patients with advanced disease. This study examined the effect of body composition on survival in primary operable GI cancer.

**Methods:**

Patients with resectable adenocarcinoma of the GI tract (esophagus, stomach, colon, rectum) between 2006 and 2014 were identified from a prospective database. Computed tomography (CT) scans were analyzed using a transverse section at L3 to calculate sex-specific body composition indices for skeletal muscle, visceral fat, and subcutaneous fat. Kaplan–Meier and log-rank analysis were used to compare unadjusted survival. Multivariate survival analyses were performed using a proportional hazards model.

**Results:**

The study enrolled 447 patients (191 woman and 256 men) with esophagogastric (OG) (*n* = 108) and colorectal (CR) (*n* = 339) cancer. Body composition did not predict survival for the OG cancer patients. Among the CR cancer patients, survival was shorter for those with sarcopenia (*p* = 0.017) or low levels of subcutaneous fat (*p* = 0.005). Older age (*p* = 0.046) and neutrophilia (*p* = 0.013) were associated with sarcopenia in patients with CR. Tumor stage (*p* = 0.033), neutrophil count (*p* = 0.011), and hypoalbuminemia (*p* = 0.023) were associated with sarcopenia in OG cancer patients. In the multivariate analysis, no single measure of body composition was an independent predictor of reduced survival.

**Conclusion:**

Sarcopenia and reduced subcutaneous adiposity are associated with reduced survival for patients with primary operable CR cancer. However, in this study, no parameter of body composition was an independent prognostic marker when considered with age, tumor stage, and systemic inflammation.

An increasing number of reports have suggested that body composition parameters may be used to predict outcomes for patients with cancer.^1^–^7^ In particular, depletion of skeletal muscle mass, termed “sarcopenia,” is widely reported to confer a poor prognosis for patients with tumors of the gastrointestinal (GI) tract, associated with an increased rate of postoperative complications^2^ and impaired response to chemotherapy.^1^ A smaller number of studies also have reported relationships between subcutaneous or visceral adiposity and outcomes for several tumor types, including esophageal,^8^ pancreatic,^9^ and colorectal cancers.^10^,^11^ The majority of these studies have used image analysis of computed tomography (CT) scans to measure parameters of body composition, and the accuracy of this technique is now widely accepted.^12^ This approach has considerable practical appeal because most patients with GI cancers undergo CT scanning as part of routine staging.

Despite consistent reports regarding short-term outcomes, the evidence that body composition parameters relate to long-term survival for patients with GI cancers has been conflicting. Studies to date have tended to focus exclusively on one parameter of body composition such as skeletal muscle mass,^3^,^4^,^6^ and much of the survival data has been derived from small cohorts of patients with locally advanced or metastatic disease.^4^,^6^,^7^

To investigate this topic further, the current study aimed to analyze CT-measured parameters of body composition in a large cohort of patients with primary operable GI cancers and to examine their relationships with long-term survival.

## Methods

Patients with confirmed adenocarcinoma of the gastrointestinal tract (esophagus, stomach, colon, and rectum) who underwent surgical resection with curative intent between 1 January 2006 and 31 December 2014 at Aberdeen Royal Infirmary were identified from a prospectively maintained regional database. Of these patients, only those who had preoperative CT images stored in an electronic format suitable for image analysis were included in the study.

All tumors were confirmed histologically and staged according to conventional American Joint Committee on Cancer (AJCC) Tumor, Node, and Metastases (TNM) Classification (6th edition). Additional pathologic data, including the presence or absence of lymphovascular invasion, were recorded from reports issued at the time of resection.

Patient variables recorded retrospectively from medical records included age, sex, and preoperative blood results recorded within 30 days before surgery. Using local reference values, anemia was defined as hemoglobin concentrations lower than 130 g/L in males and lower than 115 g/L in females. The systemic inflammatory response was assessed by differential serum white cell count (total white cell count, neutrophil count, and lymphocyte count) in line with published thresholds.^13^,^14^

The standard oncologic treatment for potentially resectable esophagogastric (OG) cancers was three cycles of neoadjuvant combination chemotherapy with epirubicin, cisplatin and capecitabine (ECX), followed by surgical resection and adjuvant chemotherapy with the same agents. Colon cancer was generally managed by surgical resection followed by adjuvant combination (fluorouracil- and oxaliplatin-based) chemotherapy for patients with involved lymph nodes or other pathologic indicators of a poor prognosis such as extramural venous invasion (EMVI). Locally advanced or margin-threatened rectal cancer was treated with “long course” chemoradiotherapy followed by surgery 8–10 weeks later, with adjuvant chemotherapy offered selectively for those with a good or partial response to preoperative treatment. Individual regimens changed over time and were dependent on patient fitness, inclusion in contemporary clinical trials, and multidisciplinary team (MDT) preference.

To perform the body composition analysis, staging computed tomography (CT) scans were first accessed through the hospital’s Picture Archiving and Communication System (PACS). Preoperative staging CTs before the start of neoadjuvant therapy were selected. A single slice at the level of the third lumbar vertebra (L3) was analyzed using medical imaging software (ImageJ; The National Institutes of Health, Washington, MD, USA; version 1.47), and the total fat area (cm^2^), subcutaneous fat area (cm^2^), visceral fat area (cm^2^), and skeletal muscle area (cm^2^) were measured using accepted Hounsfield unit (HU) thresholds (adipose tissue, −190 to −30; skeletal muscle, −29 to +150). Finally, each parameter was normalized for patient stature and designated as total fat index (cm^2^/m^2^), subcutaneous fat index (cm^2^/m^2^), visceral fat index (cm^2^/m^2^), and skeletal muscle index (cm^2^/m^2^) in line with accepted methodology.^15^,^16^ Sarcopenia was defined as a skeletal muscle index lower than 43 cm^2^/m^2^ for males and lower than 41 cm^2^/m^2^ for females using previously published cutoff values.^6^

The primary end point of the study was overall survival, which was measured in months from the date of surgery to the date of death from any cause. The date of death was obtained from patients’ electronic medical records. All survival analyses were performed after exclusion of 30-day postoperative deaths. Ethical guidance was sought from the regional Caldicott Guardian, who confirmed that the study fulfilled the criteria of a clinical audit, negating the requirement for further ethical committee approval.

### Statistical Analysis

All variables were grouped according to clinically relevant or previously published thresholds. All statistical tests were two-sided, and a *p* value lower than 0.05 was considered to indicate statistical significance. *χ* ^2^ and Mann–Whitney *U* tests were used to compare clinical characteristics between groups. Kaplan–Meier analysis and the log-rank test were used to compare unadjusted survival differences. Uni- and multivariate survival analyses were performed using a Cox proportional hazards model. Statistical analysis was performed using SPSS, version 22 (SPSS, Chicago, IL, USA).

## Results

**Fig. 1:**
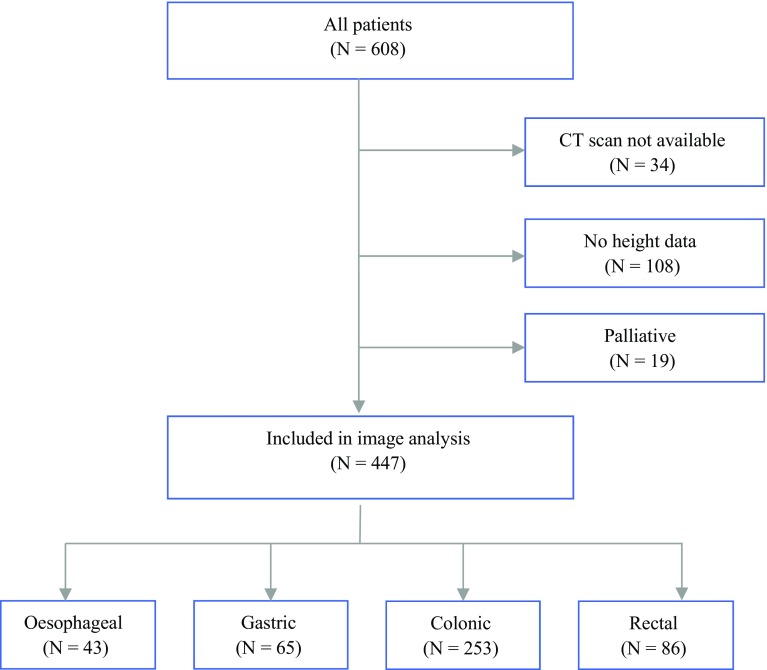
Flow diagram showing patient selection and reasons for exclusion of patients from the study

During the study period, 608 patients with primary operable gastrointestinal cancers who had undergone surgical resection with curative intent were identified. Of these patients, 161 were excluded from the study (108 patients did not have a documented height and weight; 34 patients did not have CT images suitable for analysis; and 19 patients underwent a palliative procedure after more extensive disease had been diagnosed intraoperatively), leaving 447 patients (191 women and 256 men) included in the final analysis. A flow diagram of the study selection process is shown in Fig. 1.

The baseline clinicopathologic characteristics and body composition parameters of the cohort are shown in Table 1. Of the 447 patients included in the study, 108 had eophagogastric (OG) cancers (43 esophageal; 65 gastric), and 339 had colorectal (CR) cancers (253 colonic; 86 rectal). More than 40% of the patients were anemic preoperatively, and 18% exhibited a systemic inflammatory response, as evidenced by an elevated neutrophil count. There were significant differences between upper GI and colorectal cancer in terms of age (*p* < 0.001), sex (*p* = 0.003), and lymphovascular invasion (*p* < 0.001).

**Table 1:** Clinical, pathologic, and body composition parameters of the included patients

Variable	All patients (*n* = 447) *n* (%)	OG cancer *n* (%)	CR cancer *n* (%)	*p* value^a^
Age (years)				
≤65	133 (30)	46 (43)	87 (26)	<0.001
65–74	148 (33)	40 (37)	108 (32)	
≥75	166 (37)	22 (20)	144 (42)	
Sex				
Female	191 (43)	33 (31)	158 (47)	0.003
Male	256 (57)	74 (69)	181 (53)	
Neoadjuvant therapy				
No	316 (71)	43 (40)	273 (81)	<0.001
Yes	131 (29)	65 (60)	66 (19)	
Adjuvant therapy				
No	343 (77)	66 (61)	277 (82)	<0.001
Yes	104 (23)	42 (39)	62 (18)	
TNM stage				
1	88 (20)	30 (28)	58 (17)	0.052
2	196 (44)	43 (40)	153 (45)	
3	163 (36)	35 (32)	128 (38)	
Lymphovascular invasion				
Yes	111 (25)	51 (47)	60 (18)	<0.001
No	336 (75)	57 (53)	279 (82)	
Anemia^b,c^				
Yes	186 (42)	44 (42)	142 (42)	0.873
No	255 (58)	62 (58)	193 (58)	
White cell count (× 10^9^/L)^c^				
<8.5	280 (63)	70 (66)	210 (63)	0.711
8.5–11	109 (25)	23 (22)	86 (26)	
>11	52 (12)	13 (12)	39 (12)	
Neutrophil count (× 10^9^/L)^c^				
<7.5	362 (82)	87 (82)	275 (82)	0.997
≥7.5	79 (18)	19 (18)	60 (18)	
Lymphocyte count (× 10^9^/L)^c^				
<1.0	94 (21)	18 (17)	76 (23)	0.211
≥1.0	347 (79)	88 (83)	259 (77)	
Albumin (g/L)^c^				
≥35	387 (88)	89 (84)	298 (89)	0.172
<35	54 (12)	17 (16)	37 (11)	
Subcutaneous fat index (cm^2^/m^2^)				
Median	66.2	64.9	70.0	0.114
Range	200.5	193.4	191.9	
Low ^d^	152 (34)	38 (35)	114 (34)	
Medium^d^	148 (33)	33 (31)	115 (34)	
High^d^	147 (33)	37 (34)	110 (32)	
Visceral fat index (cm^2^/m^2^)				
Median	61.3	63.4	61.0	0.886
Range	198.4	155.0	198.4	
Low^e^	152 (34)	38 (35)	114 (34)	
Medium^e^	146 (33)	38 (35)	108 (32)	
High^d^	149 (33)	32 (30)	117 (35)	
Skeletal muscle index (cm^2^/m^2^)				
Median	47.4	47.7	47.3	0.888
Range	80.1	44.2	80.1	
Sarcopenia^f^	104 (23)	23 (21)	81 (24)	
Normal	343 (77)	85 (79)	258 (76)	

*OG* esophagogastric, *CR* colorectal, *TNM* tumor-node-metastasis

^a^ *p* Values represent *X* ^2^ tests for a linear trend in categorical variables and Mann–Whitney *U* tests for continuous variables

^b^Anemia is defined as <13 g/d*L* in males, <11.5 g/d*L* in females

^c^Data are missing in six cases

^d^Sex-specific tertiles for subcutaneous fat index

^e^Sex-specific tertiles for visceral fat index

^f^Sarcopenia is defined as <43 cm^2^/m^2^ in males and <41 cm^2^/m^2^ in females

To account for the differences in body composition distribution between the men and women, the subcutaneous fat index and the visceral fat index were classified into sex-specific tertiles, whereas previously published sex-specific cutoff values for skeletal muscle index were used to define sarcopenia in the men (<43 cm^2^/m^2^) and the women (<41 cm^2^/m^2^). According to these definitions, 23 patients (21%) with esophagogastric cancer and 81 patients (24%) with colorectal cancer showed evidence of sarcopenia on their staging CT scan (Table 1).

**Fig. 2:**
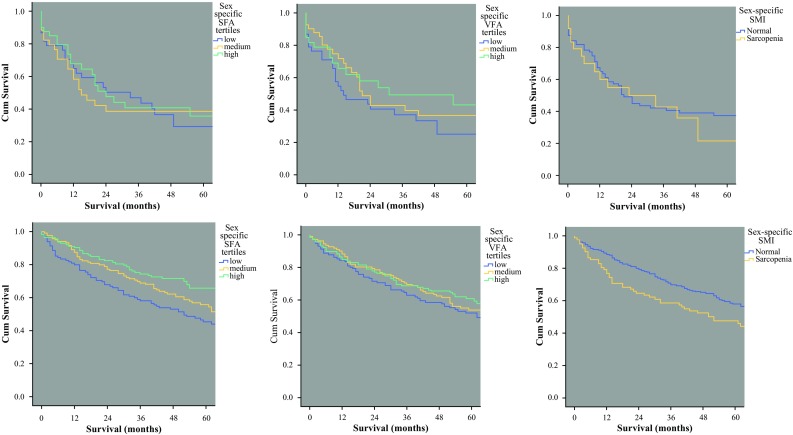
The relationships between body composition parameters and overall survival for patients with primary operable gastrointestinal cancers. *Top panel* (*left* to* right*): subcutaneous fat index (SFA) (*p* = 0.793, log-rank test), visceral fat index (VFA) (*p* = 0.278, log-rank test), and skeletal muscle index (SMI; sarcopenia) (*p* = 0.607, log-rank test) in esophagogastric cancer. *Bottom panel* (*left* to* right*): subcutaneous fat index (SFA) (*p* = 0.005, log-rank test), visceral fat index (VFA) (*p* = 0.375, log-rank test), and skeletal muscle index (SMI; sarcopenia) (*p* = 0.017, log-rank test) in colorectal cancer

Figure 2 shows the relationships between body composition parameters and long-term survival. Levels of subcutaneous fat, visceral fat, and skeletal muscle did not influence overall survival for the patients with esophagogastric cancer. Among the patients with colorectal cancer, survival was significantly shorter for those with low levels of subcutaneous fat (*p* = 0.005, log-rank test) or evidence of sarcopenia (*p* = 0.017, log-rank test).

To investigate these relationships further, the associations between body composition and clinicopathologic variables were examined. An association between sarcopenia and advanced T stage (*p* = 0.033), elevated neutrophil count (*p* = 0.011), and hypoalbuminemia (*p* = 0.023) was observed in the patients with esophagogastric cancer (Table 2). In the patients with colorectal cancer, associations between sarcopenia and older age (*p* = 0.046) and elevated neutrophil count (*p* = 0.026) were demonstrated. Similar relationships were seen between low levels of subcutaneous fat and older age (*p* < 0.001) and elevated neutrophil count (*p* = 0.013) (Table 3).

**Table 2:** Associations between body composition parameters and clinicopathologic variables for patients with esophagogastric cancer

Variable	Subcutaneous fat index			*p* value^a^	Visceral fat index			*p* value^a^	Skeletal muscle index		*p* value^a^
	Low *n* (%)	Medium *n* (%)	High *n* (%)		Low *n* (%)	Medium *n* (%)	High *n* (%)		Normal *n* (%)	Sarcopenia *n* (%)	
Age (years)											
≤64	13 (34)	15 (45)	18 (49)	0.560	20 (53)	14 (37)	12 (38)	0.533	37 (44)	9 (39)	0.744
65–74	16 (42)	10 (30)	14 (38)		11 (29)	17 (45)	12 (38)		32 (38)	8 (35)	
≥75	9 (24)	8 (24)	5 (14)		7 (18)	7 (18)	8 (25)		16 (19)	6 (26)	
Tumour (T) stage											
0/1	8 (21)	5 (15)	9 (24)	0.742	6 (16)	7 (18)	9 (28)	0.534	21 (25)	1 (4)	0.033
2	4 (11)	9 (27)	5 (14)		5 (13)	9 (24)	4 (13)		13 (15)	5 (22)	
3	22 (58)	17 (52)	20 (54)		23 (61)	18 (47)	18 (56)		47 (55)	12 (52)	
4	4 (11)	2 (6)	3 (8)		4 (11)	4 (11)	1 (3)		4 (5)	5 (22)	
Nodal (N) stage											
0	17 (45)	13 (39)	20 (54)	0.776	13 (34)	15 (39)	22 (69)	0.103	41 (48)	9 (39)	0.362
1	12 (32)	13 (39)	10 (27)		14 (37)	15 (39)	6 (19)		29 (34)	6 (26)	
2	9 (24)	7 (21)	7 (19)		11 (29)	8 (21)	4 (13)		15 (18)	8 (35)	
TNM stage											
I	9 (24)	10 (30)	11 (30)	0.846	6 (16)	11 (29)	13 (41)	0.136	27 (32)	3 (13)	0.175
II	16 (42)	11 (33)	16 (43)		15 (39)	16 (42)	12 (38)		33 (39)	10 (43)	
III	13 (34)	12 (36)	10 (27)		17 (45)	11 (29)	7 (22)		25 (29)	10 (43)	
Neoadjuvant therapy											
Yes	18 (47)	8 (24)	17 (46)	0.090	9 (24)	20 (53)	14 (44)	0.031	34 (40)	9 (39)	0.940
No	20 (53)	25 (76)	20 (54)		29 (76)	18 (47)	18 (56)		51 (60)	14 (61)	
Adjuvant therapy											
Yes	17 (45)	10 (30)	15 (41)	0.446	18 (47)	13 (34)	11 (34)	0.412	30 (35)	12 (52)	0.141
No	21 (55)	23 (70)	22 (59)		20 (53)	25 (66)	21 (66)		55 (65)	11 (48)	
Lymphovascular invasion											
Yes	17 (45)	13 (39)	21 (57)	0.324	21 (55)	16 (42)	14 (44)	0.463	38 (45)	13 (57)	0.314
No	21 (55)	20 (61)	16 (43)		17 (45)	22 (58)	18 (56)		47 (55)	10 (43)	
Anemia^b^											
Yes	17 (45)	14 (45)	13 (35)	0.621	19 (50)	11 (30)	14 (45)	0.181	33 (39)	11 (50)	0.364
No	21 (55)	17 (55)	24 (65)		19 (50)	26 (70)	17 (55)		51 (61)	11 (50)	
White cell count (×10^9^/L)											
<8.5	23 (61)	21 (68)	26 (70)	0.840	24 (63)	23 (62)	23 (74)	0.736	61 (73)	9 (41)	0.011
8.5–11	10 (26)	7 (23)	6 (16)		8 (21)	10 (27)	5 (16)		16 (19)	7 (32)	
>11	5 (13)	3 (10)	5 (14)		6 (16)	4 (11)	3 (10)		7 (8)	6 (27)	
Neutrophil count (×10^9^/L)											
<7.5	29 (76)	26 (84)	32 (86)	0.493	30 (79)	31 (84)	26 (84)	0.821	73 (87)	14 (64)	0.011
≥7.5	9 (24)	5 (16)	5 (14)		8 (21)	6 (16)	5 (16)		11 (13)	8 (36)	
Lymphocyte count (× 10^9^/L)											
<1.0	7 (18)	6 (19)	5 (14)	0.781	7 (18)	6 (16)	5 (16)	0.957	14 (17)	4 (18)	0.866
≥1.0	31 (82)	25 (81)	32 (86)		31 (82)	31 (84)	26 (84)		70 (83)	18 (82)	
Albumin (g/L)											
≥35	31 (82)	24 (77)	34 (92)	0.238	31 (82)	30 (81)	28 (90)	0.517	74 (88)	15 (68)	0.023
<35	7 (18)	7 (23)	3 (8)		7 (18)	7 (19)	3 (10)		10 (12)	7 (32)	

*TNM* tumor-node-metastasis

^a^ *p* Values represent *X* ^2^ tests for a linear trend in categorical variables and Mann–Whitney *U* tests for continuous variables

^b^Anemia is defined as <13 g/d*L* in males, <11.5 g/d*L* in females

**Table 3:** The associations between body composition parameters and clinicopathologic variables in patients with colorectal cancer

Variable	Subcutaneous fat index			*p* value^a^	Visceral fat index			*p* value^a^	Skeletal muscle index		*p* Value^a^
	Low *n* (%)	Medium *n* (%)	High *n* (%)		Low *n* (%)	Medium *n* (%)	High *n* (%)		Normal *n* (%)	Sarcopenia *n* (%)	
Age (years)											
≤64	25 (22)	23 (20)	39 (35)	<0.001	43 (38)	21 (19)	23 (20)	<0.001	70 (27)	17 (21)	0.046
65–74	27 (23)	39 (34)	42 (38)		18 (16)	39 (36)	51 (44)		88 (34)	20 (25)	
≥75	62 (54)	53 (46)	29 (26)		53 (46)	48 (44)	43 (37)		100 (39)	44 (54)	
Tumour (T) stage											
0/1	9 (8)	5 (4)	8 (7)	0.432	7 (6)	10 (9)	5 (4)	0.219	17 (7)	5 (6)	0.118
2	12 (11)	13 (11)	20 (18)		13 (11)	13 (12)	19 (16)		40 (16)	5 (6)	
3	72 (63)	76 (66)	69 (63)		73 (64)	74 (69)	70 (60)		164 (64)	53 (65)	
4	21 (18)	21 (18)	13 (12)		21 (18)	11 (10)	23 (20)		37 (14)	18 (22)	
Nodal (N) stage											
0	69 (61)	65 (57)	77 (70)	0.099	69 (61)	62 (57)	80 (68)	0.482	168 (65)	43 (53)	0.099
1	24 (21)	35 (30)	19 (17)		29 (25)	27 (25)	22 (19)		57 (22)	21 (26)	
2	21 (18)	15 (13)	14 (13)		16 (14)	19 (18)	15 (13)		33 (13)	17 (21)	
TNM stage											
1	19 (17)	13 (11)	26 (24)	0.094	16 (14)	18 (17)	24 (21)	0.398	50 (19)	8 (10)	0.058
2	50 (44)	52 (45)	51 (46)		53 (46)	44 (41)	56 (48)		118 (46)	35 (43)	
3	45 (39)	50 (43)	33 (30)		45 (39)	46 (43)	37 (32)		90 (35)	38 (47)	
Neoadjuvant therapy											
Yes	89 (78)	92 (80)	92 (84)	0.566	88 (77)	88 (81)	97 (83)	0.524	204 (79)	69 (85)	0.225
No	25 (22)	23 (20)	18 (16)		26 (23)	20 (19)	20 (17)		54 (21)	12 (15)	
Adjuvant therapy											
Yes	15 (13)	22 (19)	25 (23)	0.173	26 (23)	16 (15)	20 (17)	0.281	49 (19)	13 (16)	0.550
No	99 (87)	93 (81)	85 (77)		88 (77)	92 (85)	97 (83)		209 (81)	68 (84)	
Lymphovascular invasion											
Yes	23 (20)	20 (17)	17 (15)	0.648	20 (18)	18 (17)	22 (19)	0.914	45 (17)	15 (19)	0.825
No	91 (80)	95 (83)	93 (85)		94 (82)	90 (83)	95 (81)		213 (83)	66 (81)	
Anemia^b^											
Yes	53 (47)	53 (46)	36 (33)	0.069	49 (44)	44 (41)	49 (42)	0.925	105 (41)	37 (46)	0.423
No	60 (53)	61 (54)	72 (67)		63 (56)	63 (59)	67 (58)		150 (59)	43 (54)	
White cell count (×10^9^/L)											
<8.5	64 (57)	73 (64)	73 (68)	0.241	76 (68)	64 (60)	70 (60)	0.110	162 (64)	48 (60)	0.561
8.5–11	31 (27)	32 (28)	23 (21)		24 (21)	25 (23)	37 (32)		66 (26)	20 (25)	
>11	18 (16)	9 (8)	12 (11)		12 (11)	18 (17)	9 (8)		27 (11)	12 (15)	
Neutrophil count (×10^9^/L)											
<7.5	83 (73)	99 (87)	93 (86)	0.013	94 (84)	85 (79)	96 (83)	0.669	216 (85)	59 (74)	0.026
≥7.5	30 (27)	15 (13)	15 (14)		18 (16)	22 (21)	20 (17)		39 (15)	21 (26)	
Lymphocyte count (×10^9^/L)											
<1.0	31 (27)	23 (20)	22 (20)	0.334	28 (25)	29 (27)	19 (16)	0.125	57 (22)	19 (24)	0.795
≥1.0	82 (73)	91 (80)	86 (80)		84 (75)	78 (73)	97 (84)		198 (78)	61 (76)	
Albumin (g/L)											
≥35	97 (86)	101 (89)	100 (93)	0.275	98 (88)	95 (89)	105 (91)	0.766	229 (90)	69 (86)	0.376
<35	16 (14)	13 (11)	8 (7)		14 (13)	12 (11)	11 (9)		26 (10)	11 (14)	

TNM, tumor-node-metastasis

^a^ *p* values represent *X* ^2^ tests for a linear trend in categorical variables and Mann–Whitney *U* tests for continuous variables

^b^Anemia is defined as <13 g/d*L* in males, <11.5 g/d*L* in females

Finally, logistic regression analyses were used to examine whether survival relationships were independent of established clinicopathologic risk factors. During the follow-up period, 213 patients died, leaving 234 were alive at the date of censor (31 March 2015). The median follow-up period for the survivors was 62 months (range 3–105 months).

In the multivariate analysis, the only independent predictor of long-term survival for the patients with esophagogastric cancer was tumor stage [hazard ratio (HR) 2.78; *p* < 0.001] (Table 4). For the patients with colorectal cancer, advanced tumor stage (HR 1.67; *p* < 0.001), lymphovascular invasion (HR 2.61; *p* < 0.001), and elevated neutrophil count (HR 1.76; *p* = 0.005) were independently associated with reduced overall survival (Table 5). No single measure of body composition was an independent predictor of reduced survival for patients with primary operable GI cancer.

**Table 4:** Multivariate analysis of the relationships between body composition parameters and overall survival for patients with esophagogastric cancer

Variables	No. of patients	No. of deaths *n* (%)	Univariate analysis			Multivariate analysis		
			HR	(95% CI)	*p* value	HR	(95% CI)	*p* value
Age (years)								
≤65	46	23 (33)	1.148	0.832–1.584	0.402	1.578	1.03–2.417	NS
65–74	40	27 (40)						
≥75	22	12 (35)						
Sex								
Female	33	18 (35)	1.026	0.593–1.777	0.926	1.145	0.605–2.167	0.667
Male	75	44 (37)						
TNM stage								
1	30	5 (14)	2.390	1.681–3.398	<0.001	2.782	1.766–4.382	<0.001
2	43	30 (41)						
3	35	27 (44)						
Neoadjuvant therapy								
Yes	43	20 (32)	1.579	0.926–2.691	0.093	2.111	1.015–4.388	NS
No	65	42 (39)						
Adjuvant therapy								
Yes	42	23 (35)	0.719	0.429–1.206	0.719	0.403	0.22–0.737	NS
No	66	39 (37)						
Lymphovascular invasion								
Yes	51	34 (40)	1.722	1.037–2.859	0.036	0.814	0.425–1.560	NS
No	57	28 (33)						
Neutrophil count (×10^9^/L)								
<7.5	87	48 (36)	1.033	0.549–1.946	0.919	1.048	0.517–2.124	NS
≥7.5	19	12 (39)						
Subcutaneous fat index (cm^2^/m^2^)								
High	38	24 (39)	0.912	0.678–1.228	0.545	0.934	0.627–1.39	NS
Medium	33	19 (37)						
Low	37	19 (34)						
Visceral fat index (cm^2^/m^2^)								
High	38	26 (41)	0.786	0.571–1.083	0.141	0.738	0.473–1.152	NS
Medium	38	21 (36)						
Low	32	15 (32)						
Skeletal muscle index (cm^2^/m^2^)								
Normal	85	48 (36)	1.165	0.642–2.114	0.616	0.761	0.351–1.649	NS
Sarcopenia	23	14 (38)						

*HR* hazard ratio, *CI* confidence interval, *TNM* tumor-node-metastasis

**Table 5:** Multivariate analysis of the relationships between body composition parameters and overall survival for patients with colorectal cancer

Variable	No. of patients	No. of deaths *n* (%)	Univariate analysis			Multivariate analysis		
			HR	(95% CI)	*p* value	HR	(95% CI)	*p* Value
Age (years)								
≤65	87	36 (29)	1.197	0.976–1.467	0.084	1.099	0.871–1.386	NS
65–74	108	41 (28)						
≥75	144	74 (34)						
Sex								
Female	158	74 (32)	0.856	0.622–1.176	0.339	0.994	0.703–1.405	NS
Male	181	77 (30)						
TNM stage								
1	58	12 (17)	1.921	1.503–2.455	<0.001	1.667	1.263–2.2	<0.001
2	153	64 (29)						
3	128	75 (37)						
Neoadjuvant therapy								
Yes	273	120 (31)	1.095	0.738–1.626	0.651	1.444	0.946–2.203	NS
No	66	31 (32)						
Adjuvant therapy								
Yes	62	28 (31)	0.979	0.649–1.476	0.976	0.764	0.479–1.218	NS
No	277	123 (31)						
Lymphovascular invasion								
Yes	60	48 (44)	3.663	2.585–5.190	<0.001	2.606	1.764–3.851	<0.001
No	279	103 (27)						
Neutrophil count (× 10^9^/L)								
<7.5	275	108 (28)	2.556	1.780–3.669	<0.001	1.760	1.182–2.62	0.005
≥7.5	60	41 (41)						
Subcutaneous fat index (cm^2^/m^2^)								
High	114	62 (35)	0.720	0.589–0.880	0.001	0.846	0.662–1.08	NS
Medium	115	52 (31)						
Low	110	37 (25)						
Visceral fat index (cm^2^/m^2^)								
High	114	56 (33)	0.873	0.718–1/061	0.172	1.00	0.796–1.256	NS
Medium	108	48 (31)						
Low	117	47 (29)						
Skeletal muscle index (cm^2^/m^2^)								
Normal	258	107 (29)	1.527	1.075–2.170	0.018	1.211	0.818–1.795	NS
Sarcopenia	81	44 (35)						

*HR* hazard ratio, *CI* confidence interval, *TNM* tumor-node-metastasis

## Discussion

The results of the current study show that CT measures of body composition, particularly sarcopenia and reduced levels of subcutaneous fat, are associated with shorter survival for patients with primary operable colorectal cancer, but not for patients with esophagogastric cancer. Furthermore, strong associations exist between these parameters and other indicators of poor outcome such as advanced age and elevated systemic inflammatory response. However, when body composition parameters were analyzed in a multivariate model, no single measure was found to have independent predictive value for patients with either esophagogastric or colorectal cancer.

To our knowledge, this is one of the largest studies to investigate the impact of body composition on long-term survival of patients with operable GI cancers. Although associations between sarcopenia and colorectal cancer outcomes have been reported previously,^3^,^4^,^6^,^7^,^17^,^18^ the results have been inconsistent. Most previous studies have included a high proportion of patients with advanced disease, whereas the current study focused specifically on patients with operable disease.

A systematic review by Malietzis et al.^2^ evaluated the role of body composition in predicting outcomes for patients with colorectal cancer and concluded that whereas evidence was consistent that sarcopenia is associated with poorer short-term outcomes, including excess chemotherapy toxicity^17^–^19^ and an increased risk of surgical complications,^20^,^21^ the evidence for a relationship with long-term survival was less robust. Indeed, the reviewers identified only one study of 196 patients, all of whom had metastatic disease,^7^ in which sarcopenia had a detrimental effect on survival.

Not included in the aforementioned review but widely referenced as demonstrating the prognostic value of skeletal muscle depletion for cancer patients, a study by Martin et al.^6^ analyzed the body composition parameters of 1473 patients with respiratory and GI cancers. The authors reported that a predictive model composed entirely of body composition variables (weight loss, skeletal muscle depletion, and muscle attenuation) was superior to conventional prognostic markers, including cancer stage. However, more than 50% of the patients studied had metastatic disease, and our results suggest that their findings may not be applicable to patients with primary operable cancers.

It is clear from our own appraisal of the literature and the conclusions of recent reviews^3^,^4^ that the question whether sarcopenia has prognostic value for patients with GI malignancies is being hampered by study heterogeneity. Despite the volume of published work, there still is no standard definition of CT-based assessments of skeletal muscle mass.

Although a number of different cutoff values have been proposed,^7^,^17^,^22^ we chose to use a skeletal muscle index lower than 43 cm^2^/m^2^ for men and lower than 41 cm^2^/m^2^ for women to define sarcopenia. These values were proposed by the largest published dataset to document the body composition of patients with cancer^6^ and have been validated in at least one external cohort.^7^

It must be emphasised that discrepancies in the thresholds used to define sarcopenia have led to considerable variation in the proportion of patients reported to be “sarcopenic” in the aforementioned studies. For example, the study by van Vledder et al.,^7^ using one threshold, reported that 19% of patients with colorectal liver metastases have sarcopenia, whereas Martin et al.,^6^ using different definitions, reported that 53% of women and 31% of men are sarcopenic. Using the latter definitions, our levels of sarcopenia were considerably lower (23%), but all the patients in our cohort were undergoing curative surgery, whereas their study contained a large number of patients with metastatic disease. Similarly, the assessment of subcutaneous and visceral adiposity has been undertaken using a variety of methods including dichotomous cutoff values,^23^,^24^ continuous parameters,^25^ and visceral-to-subcutaneous ratios.^26^

Given this variability and with no single method yet validated, we chose to use sex-specific tertiles to assess adiposity. It may be that using an alternative technique would have yielded different results, but we believe our approach was a rational way of demonstrating any survival effect.

One noteworthy finding from the current study was the association between depleted levels of skeletal muscle and subcutaneous fat and an elevation of the systemic inflammatory response in patients with colorectal cancer. The neutrophil count was used as a marker of systemic inflammation because findings previously showed it to be the most reliable prognostic component of the white cell count.^27^

In experimental models, pro-inflammatory cytokines such as interleukin-1 (IL-1), IL-6, and tumor necrosis factor-α (TNF) have been shown to play a key role in both anorexia and skeletal muscle proteolysis,^28^ but the relationships between systemic inflammation and changes in body composition in cancer patients are less well understood. Good evidence currently shows that systemic inflammation is universally associated with poor short- and long-term outcomes in a variety of solid organ tumor types,^29^–^31^ and an association with skeletal muscle wasting may offer one explanation for the unfavorable outcomes observed in sarcopenic patients.^14^,^32^,^33^ In the current study, despite no significant difference in the prevalence of sarcopenia between cancer types, a clear relationship was demonstrated between sarcopenia and survival in colorectal cancer but not in upper GI cancers. Further work is needed to clarify the relationships between tumor biology, inflammatory mediators, and parameters of body composition.

The current study had a number of limitations. The retrospective nature of the data collection meant that contemporary records of patients’ height were missing in a number of cases. As a result, body composition indices could not be normalized for stature, thereby limiting the size of the cohort. Similarly, preoperative weight was poorly documented in the medical notes, so conventional parameters of body composition such as body mass index (BMI) could not be calculated. However, preoperative CT images were available for almost all the patients, and we believe that both the size and maturity of the cohort mean our results are likely to be reliable.

In summary, the current study showed that sarcopenia and reduced subcutaneous adiposity are associated with shorter overall survival for patients with primary operable colorectal cancer. However, no parameter of body composition was an independent prognostic marker when considered with age, tumor stage, and systemic inflammatory response. No relationships between body composition and overall survival were observed in patients with esophagogastric cancers.
